# Engagement, acceptability, usability and satisfaction with *Active for Life,* a computer-tailored web-based physical activity intervention using Fitbits in older adults

**DOI:** 10.1186/s12966-023-01406-4

**Published:** 2023-02-14

**Authors:** Stephanie J. Alley, Stephanie Schoeppe, Quyen G. To, Lynne Parkinson, Jannique van Uffelen, Susan Hunt, Mitch J. Duncan, Anthony Schneiders, Corneel Vandelanotte

**Affiliations:** 1 Appleton Institute, School of Health, Medical and Applied Sciences, Central Queensland University, Rockhampton, QLD Australia; 2 School of Medicine & Public Health, College of Health, Medicine, and Wellbeing, The University of Newcastle, Callaghan, NSW Australia; 3 Department of Movement Sciences, KU Leuven, Leuven, Belgium; 4 School of Nursing, Midwifery and Social Sciences, Central Queensland University, Melbourne, VIC Australia; 5 School of Health, Medical and Applied Sciences, Central Queensland University, Gladstone, QLD Australia

**Keywords:** Internet, Online, Activity trackers, Activity monitors, Wearables, Older adults, Physical activity

## Abstract

**Background:**

Preliminary evidence suggests that web-based physical activity interventions with tailored advice and Fitbit integration are effective and may be well suited to older adults. Therefore, this study aimed to examine the engagement, acceptability, usability, and satisfaction with ‘*Active for Life*,’ a web-based physical activity intervention providing computer-tailored physical activity advice to older adults.

**Methods:**

Inactive older adults (*n* = 243) were randomly assigned into 3 groups: 1) tailoring + Fitbit, 2) tailoring only, or 3) a wait-list control. The tailoring + Fitbit group and the tailoring-only group received 6 modules of computer-tailored physical activity advice over 12 weeks. The advice was informed by objective Fitbit data in the tailoring + Fitbit group and self-reported physical activity in the tailoring-only group. This study examined the engagement, acceptability, usability, and satisfaction of *Active for Life* in intervention participants (tailoring + Fitbit *n* = 78, tailoring only *n* = 96). Wait-list participants were not included. Engagement (Module completion, time on site) were objectively recorded through the intervention website. Acceptability (7-point Likert scale), usability (System Usability Scale), and satisfaction (open-ended questions) were assessed using an online survey at post intervention. ANOVA and Chi square analyses were conducted to compare outcomes between intervention groups and content analysis was used to analyse program satisfaction.

**Results:**

At post-intervention (week 12), study attrition was 28% (22/78) in the Fitbit + tailoring group and 39% (37/96) in the tailoring-only group. Engagement and acceptability were good in both groups, however there were no group differences (module completions: tailoring + Fitbit: 4.72 ± 2.04, Tailoring-only: 4.23 ± 2.25 out of 6 modules, *p* = .14, time on site: tailoring + Fitbit: 103.46 ± 70.63, Tailoring-only: 96.90 ± 76.37 min in total, *p* = .56, and acceptability of the advice: tailoring + Fitbit: 5.62 ± 0.89, Tailoring-only: 5.75 ± 0.75 out of 7, *p* = .41). Intervention usability was modest but significantly higher in the tailoring + Fitbit group (tailoring + Fitbit: 64.55 ± 13.59, Tailoring-only: 57.04 ± 2.58 out of 100, *p* = .003). Participants reported that *Active for Life* helped motivate them, held them accountable, improved their awareness of how active they were and helped them to become more active. Conversely, many participants felt as though they would prefer personal contact, more detailed tailoring and more survey response options.

**Conclusions:**

This study supports web-based physical activity interventions with computer-tailored advice and Fitbit integration as engaging and acceptable in older adults.

**Trial registration:**

Australian and New Zealand Clinical Trials Registry: ACTRN12618000646246. Registered April 23 2018, https://www.anzctr.org.au/Trial/Registration/TrialReview.aspx?id=374901

## Introduction

Despite the fact that physical activity benefits healthy ageing [1], a large proportion of older adults 65 + years are not meeting the physical activity recommendations of 30 min on most days [2]. The percentage of older Australians aged 65 + years who regularly connect to the internet is high and growing (62%) [3], and web-based physical activity interventions have shown to be effective at increasing physical activity in older adults [4, 5]. Specifically, web-based computer-tailored interventions which provide personalised physical activity advice to participants have shown to be effective at increasing physical activity in adults 18 + years of age [6]. Older adults have a higher prevalence of health issues (e.g., diabetes, cancer, heart disease) and co-morbidities which are associated with physical inactivity [2]. Therefore, web-based computer-tailored interventions have much potential for older adults as they can personalise physical activity advice based on such health issues [7]. Despite this, few studies have specifically tested the effectiveness of computer-tailored physical activity advice built for adults 65 + years and those studies that have demonstrated mixed results in terms of increasing physical activity [7–9].

Computer-tailored advice is typically based on a brief survey of participants’ physical activity levels, characteristics, environment, and psychosocial correlates of physical activity [6]. However, new technology allows the physical activity component to be measured via advanced activity trackers (e.g., Fitbit) [10]. Fitbit integration has been shown to improve usability, non-usage attrition, acceptability, and effectiveness of computer tailored advice in adults 18 + years of age [10]. Interventions using activity trackers in older adults are effective [11], however no studies have tested the acceptability of Fitbit data to inform computer-tailored physical activity advice in older adults. In general, the uptake of activity trackers is lower in the older adult age group [12] and it is unknown if Fitbit integration would be well received by older adults.

Older adults may interact with computer-tailored advice and Fitbit technology differently than middle-aged adults who make up most participants in evaluations of computer-tailored interventions and activity trackers [6, 13]. More broadly, older adults are typically more reserved towards new technology and more sensitive to design [14]. Some evidence suggests that older adults demonstrate good compliance and are satisfied with using Fitbit activity trackers [15, 16]. However, there is research that indicates that non-usage attrition in web-based computer-tailored programs increases with age [17]. Currently, we do not know how older adults interact with an perceive a web-based computer-tailored physical activity intervention with Fitbit integration.

We recently conducted a randomised controlled trial to test the efficacy of a web-based physical activity intervention (*Active for Life)* providing tailored physical activity advice with and without Fitbit integration [18]. Results demonstrated that the Fitbit and tailoring group increased their Moderate to Vigorous Physical Activity (MVPA) from baseline to post-intervention by 14 min per week which was statistically significant compared to the control group who decreased their MVPA by 49 min per week, but not statistically significant compared to the tailored advice only group who decreased their MVPA by 8 min per week. The MVPA changes from baseline to post-intervention in the tailored advice only group compared to the control group were not statistically significant. There were no group differences at 6 months follow up [18]. A detailed process evaluation is needed to understand why the Fitbit integration did not improve intervention efficacy and why the tailored physical activity advice alone was not effective in comparison to a wait-list control. Understanding how participants interacted with the *Active for Life* intervention will help to explain these results and inform the development of future web-based physical activity interventions for older adults. Therefore, this study aims to examine the engagement, acceptability, usability, and satisfaction of *Active for Life,* a web-based computer-tailored physical activity intervention for older adults with and without Fitbit integration.

## Methods

### Study design

This study presents process data from the *Active for Life* intervention. The original *Active for Life* trial was a 3-group Randomised Controlled Trial where participants were randomised into one of three groups: 1) tailoring + Fitbit, 2) tailored advice only and 3) wait list control. The protocol is published elsewhere [19]. This process evaluation study of *Active for Life* included all intervention participants (tailoring + Fitbit and tailored advice only). Control participants were not included as they were not given access to the intervention until after the study. Objective website usage data (engagement) and acceptability, usability, and satisfaction data from a post-intervention survey (week 12) are analysed.

### Participants

Recruitment was conducted between April 2018 and March 2019 in Rockhampton (Regional Queensland), Bundaberg (Regional Queensland), and Adelaide (Metropolitan South Australia), Australia through Facebook advertising, university email lists, flyers and local newsletters. Eligible participants were adults aged ≥ 65 years, who were speaking English, had Internet access, basic Internet confidence and could attend two face-to-face appointments at one of the project locations. Participants had to be able to safely increase their physical activity as assessed through the Physical Activity Readiness Questionnaire [20] or approval from their General Practitioner. Participants were ineligible if they were already meeting the physical activity guidelines [21], participating in another physical activity program or had used a Fitbit in the previous 6 months.

### Intervention

The *Active for Life* intervention is a web-based program with computer-tailored advice to encourage older adults to work towards meeting the physical activity recommendations of 30 min of moderate intensity activity on at least 5 days each week including 2–3 sessions of strength and flexibility activity. The program included 6 modules of tailored advice delivered fortnightly over 12-weeks. The tailored advice is computer automated and uses participant data to select appropriate messages from a database of messages using IF–THEN algorithms (e.g., IF ‘inactive’ AND ‘low social support’ THEN ‘display message on joining a group or class’). The advice was informed by the theory of planned behaviour [22] and the social cognitive theory [23] and includes evidence-based behaviour change techniques such as self-monitoring, goal setting, action planning, habit formation and relapse prevention [24, 25]. The advice is tailored to participants’ characteristics, environment, physical activity behaviour and psychosocial correlates of physical activity (e.g., self-efficacy and social support). Each module of advice included around 10 brief sections (e.g., progress, losing weight, goal setting, exercise with arthritis). Some sections include a graph or picture. New modules could be accessed when previous modules were completed. Participants received up to 3 automatic reminder emails to complete a new session.

A feature for creating action plans was included on the website. At the end of modules 2 and 4, all participants were encouraged to use this action planning tool to guide them in setting an action plan – the what, where, when and with whom – for being active in the following fortnight. An exercise library feature was also included on the website for participants to access 4-week strength and flexibility exercise plans. These plans were written by a physiotherapist at the beginners and/or intermediate level and participants could view video demonstrations of the exercises through a link to an external website hosted by *PhysiTrack®*. Participants were encouraged to use these plans if they were not participating in at least 2 sessions of strength and flexibility activity per week and wanted a guide to do these at home.

All intervention participants in the both the tailoring + Fitbit and the tailoring only groups were given access to the same *Active for Life* intervention website including the 6 modules of computer-tailored advice, action planning tool and exercise library. Both groups completed a brief questionnaire at the start of each module to inform the computer tailored advice. The only between group difference was that the personalised physical activity advice in the tailoring + Fitbit group was based on data collected through their Fitbit, whereas in the tailoring only group the advice was based on the answers to additional questions asking them to recall how many minutes of physical activity they had completed in the past two weeks. All other questions (e.g., sitting time, self-efficacy, social support etc.) were identical.

### Procedures

Recruitment materials directed participants to a landing page on the *Active for Life* website where they completed an eligibility survey and eligible participants were invited to complete baseline assessments. Participation included completion of four online research surveys at baseline, week 6, week 12 and week 24 and wearing an accelerometer for 7 consecutive 24-h days at baseline and week 12. Participants were sent an accelerometer via the postal service and attended a baseline appointment to return the accelerometer. During the baseline appointment participants were randomly allocated to one of the three trial arms (tailoring + Fitbit, tailored advice only and wait list control) using computer-automated block randomization with block sizes of 15 and a 1:1:1 ratio. Randomisation was stratified by age (< 75 years, ≥ 75 years) and sex (male, female). They were then shown through the intervention website. Tailoring + Fitbit participants were provided with a Fitbit Flex 1 (original model) which tracks steps, distance, calories burned, active minutes, stationary time, and sleep. It has no visual display but 5 lights to indicate progress towards a step goal. Tailoring + Fitbit participants were then guided in syncing their Fitbit Flex to the intervention website. First, they set up a Fitbit account and synced their Fitbit Flex to the Fitbit app on their smartphone, tablet or to the Fitbit website through a USB dongle. They then provided Fitbit with permission for the *Active for Life* website to extract their activity minutes summary data from their Fitbit account. During the intervention participants only needed to press one button at the start of each module for *Active for Life* to extract their latest activity minutes data from Fitbit. Participants received a take home sheet with their Fitbit device which encouraged them to wear the Fitbit as much as possible (taking it off to swim and shower) and manually add any water-based activities through the Fitbit app or website. Participants were asked to make sure their Fitbit had recently synced with their Fitbit account prior to each *Active for Life* module and to record their strength, flexibility and balance exercises in each *Active for Life* module. After the 12-week intervention, participants attended another face-to-face appointment to return the week 12 accelerometer. Participants received up to 3 automatic reminder emails for each research survey. If the surveys were still incomplete after the reminders, participants were offered a $20 voucher to complete them within the next few days. Participants received a $50 voucher after completing all research surveys.

### Measures

#### Engagement with the Active for Life intervention

Intervention engagement was assessed throughout the 12-week intervention via website data (module completion, action plan completion) and Google Analytics measures (time spent on the website, website visits, time spent reading tailored advice, time spent on the exercise library feature, time spent on the action planning feature). Study attrition was measured at week 12 by non-completion of assessments.

#### Perceived usefulness of the Active for Life intervention

Perceived usefulness was measured at week 12 by asking participants how useful they found 1) the whole program, 2) the action planning tool, and 3) the library of exercises with the following response options: 1) ‘I did not use this feature at all’, 2) ‘not at all useful’, 3) ‘not very useful’, 4) ‘neutral’, 5) ‘somewhat useful’, and 6)’very useful’. The response options were collapsed into two categories 1) useful, and 2) did not use, not useful or neutral.

#### Acceptability of the Active for Life intervention

Acceptability of the tailored advice was measured at week 12 through 9 items based on past research [10] but adapted for this study. Questions included ‘the physical activity advice was a) interesting, b) credible, c) easy to understand, d) personally relevant, e) held me accountable,’ f) ‘through the advice I learned something new about my own physical activity’, g) ‘too much physical activity was provided’, h) ‘I have used the advice to become more active’, and i) ‘I have changed my opinion about physical activity because of this program.’ The items were on a 7-point Likert scale ranging from ‘strongly agree’ to ‘strongly disagree.’ The individual questions were collapsed into 2 categories for reporting and analysis: 1) agree and 2) disagree or neutral. An average acceptability rating was calculated for each participant by averaging ratings of the 9 acceptability statements (ratings of negative statements were reverse scored).

#### Usability of the Active for Life website

Website usability was measured by the 10-item valid and reliable System Usability Scale (SUS) [26] at week 12. The SUS includes 10 items on a 5-point Likert scale from ‘strongly agree’ (5) to ‘strongly disagree’ (1) (e.g., ‘I thought the system was easy to use’). Summary scores range from 0–100 with scores above 68 representing good website usability [27]. The individual questions were collapsed into 2 categories for reporting and analysis: 1) agree and 2) disagree or neutral.

#### Satisfaction of the Active for Life intervention

Participants’ satisfaction was measured through 5 open ended questions at week 12. This included, 1) ‘do you have any other comments or suggestions,’ 2) ‘what did you like most about the program,’ 3) ‘what did you like least about the program,’ 4) ‘any suggestions to improve the program’ and 5) ‘any other comments about the program.’

#### Fitbit usage

Use of the Fitbit Flex was measured at week 12 through three questions 1) ‘during the last month, how many weeks have you worn the Fitbit?’, 2) ‘on weeks where you wore the Fitbit during the last month, on average how many days per week were you wearing the Fitbit?’ and 3) ‘on days where you wore the Fitbit during the last month, on average how many hours a day were you wearing the Fitbit?’ These questions were used to determine average hours of Fitbit use per day between weeks 9 and 12. Objective data of the number of days that the Fitbit was used in the week prior was extracted from tailoring + Fitbit participants’ accounts at each *Active for Life* session. Participants with at least 10 min of active time (light, moderate or vigorous) on a day were classified as using the Fitbit. Similar cut points have been used to determine daily use in past research. For example, many studies used the cut point ≥ 1000 steps per day which is considered equivalent to ≥ 10 active minutes per day at moderate intensity [28–30]. The number and percentage of participants who used the Fitbit for at least 5 days in the week prior to each session was then calculated.

#### Fitbit acceptability

Acceptability of the Fitbit Flex device was measured at week 12 through 8 questions based on past research [10] but adapted for this study. Questions included ‘Do you think the use of an activity tracker (Fitbit) in addition to the online physical activity advice helped to a) improve the value of the advice, b) improve the credibility of the advice, c) improve the personal relevance of the advice, d) increase your awareness of how active you are, e) meet your physical activity goals, f) make you more active, g) increase your awareness of how active you are’ and h) ‘it was easy to connect with and sync Fitbit data between the *Active for Life* website and the Fitbit website’ The items were on a 7-point Likert scale ranging from ‘strongly agree’ to ‘strongly disagree.’ The individual items were collapsed into two categories for reporting: 1) agree and 2) disagree or neutral. An average Fitbit acceptability rating was calculated for each participant by averaging ratings of the 8 acceptability statements.

#### Fitbit satisfaction

Satisfaction with the Fitbit was measured through 3 open ended questions in the tailoring + Fitbit participants at week 12. This included, 1) ‘what did you like about using the Fitbit activity tracker in this study,’ 2) ‘what did you NOT like about using the Fitbit activity tracker in this study,’ and 3) ‘How could the use of the Fitbit activity tracker be improved in the physical activity intervention.’

#### Sociodemographic characteristics

Participant demographics including gender, age, location (Adelaide, Rockhampton, Bundaberg), marital status (single or married/in a relationship), height and weight (to calculate Body Mass Index [BMI]), English as main language (yes or no), education level (primary, secondary, tech college or university), employment (fulltime, part time or not working), pre-tax household income (< A$41,599, A$41,000–64,999, A$65,000–103,999 or A$104,000 +) and chronic disease diagnosis (yes or no) were measured at baseline. Frequency of internet use (one to several times a week, once a day, several times a day) was measured at baseline in addition to internet self-efficacy assessed via the valid and reliable Internet self-efficacy scale [31]. The Internet self-efficacy scale includes 8 items of internet skills on a 7-point Likert scale of ‘strongly agree’ (7) to ‘strongly disagree’ (1). Items were added together to produce a summary score where higher scores indicate higher internet self-efficacy.

### Data analysis

Analyses were conducted using SPSS version 26 with a significance level of 0.05. One-way Analysis of Variances (ANOVA) were conducted to compare intervention groups on each continuous engagement (usage) measure. A Generalised linear model with a multinominal distribution and cumulative logit link was conducted to compare intervention groups on number of action plans completed (0, 1, or 2). Chi-square tests were used to compare intervention groups (tailoring + Fitbit and tailoring only) on the percentage of participants that perceived 1) the whole program, 2) the action planning tool, and 3) the library of exercises as useful. Chi-square analyses were conducted to compare intervention groups on the percentage of participants agreeing to each individual question on advice acceptability. A one-way ANOVA was conducted to compare intervention groups on the total advice acceptability score. Chi-square analyses were conducted to compare the intervention groups on the percentage of participants agreeing to each individual question on the SUS scale. A one-way ANOVA was conducted to compare intervention groups on the total SUS score. Content analysis was used to analyse the qualitative data responses to the opened ended questions on the *Active for Life* program and Fitbit device. Emergent themes were identified and presented in a pen profile diagram for the program (Fitbit + tailoring and tailoring only participants) and Fitbit device (Fitbit + tailoring participants) separately. A pen profile presents analysed text data in a diagram and is an increasingly utilized technique for evaluation text data [32, 33].

## Results

At post-intervention (week 12) overall study attrition was 28% (56/78) in the Fitbit + tailoring group and 39% (59/96) in the tailoring only group. Most participants accessed the website through a desktop (*n* = 60, 35.9%), a mix of devices (*n* = 58, 34.7%) or a tablet (*n* = 39, 23.4%). Only a small percentage of participants solely accessed the website through a smart phone (*n* = 10, 6%).

Table 1 shows baseline participant characteristics for both intervention groups. In all intervention participants, 79% were female, 58% were from Adelaide, 95% spoke English as their primary language, 69% were married or in a relationship, 51% had a university education, 74% were not working, 35% had a chronic disease, 74% used the internet several times a day and 46% had a household income under 40,000. The average age was 69 years. BMI was 29 kg/m^2^ (overweight) and Internet self-efficacy was good at 44 out of 56 points [31].

**Table 1:** Baseline participant characteristics

Baseline Characteristics	All intervention participants (*n* = 174)	Tailoring + Fitbit (*n* = 78)	Tailoring only (*n* = 96)
**Sex n (%)**			
Male	37 (21.3)	18 (23.1)	19 (19.8)
Female	137 (78.7)	60 (76.9)	77 (80.2)
**Location, n (%)**			
Rockhampton	60 (34.5)	24 (30.8)	36 (37.5)
Bundaberg	13 (7.5)	8 (10.3)	5 (5.2)
Adelaide	101 (58.0)	46 (59.0)	55 (57.3)
**Primary language, n (%)**			
English	166 (95.4)	72 (92.3)	94 (97.9)
Other	8 (4.6)	6 (7.7)	2 (2.1)
**Marital status, n (%)**			
Single	54 (31.0)	22 (28.2)	32 (33.3)
Married/in a relationship	120 (69.0)	56 (71.8)	64 (66.7)
**Education, n (%)**			
Secondary School	46 (26.4)	25 (32.1)	21 (21.8)
Technical collage	39 (22.4)	11 (14.1)	29 (29.2)
University	89 (51.1)	42 (53.8)	47 (49.0)
**Employment, n (%)**			
Full time	15 (8.6)	8 (10.3)	7 (7.3)
Part time or casual	31 (17.8)	16 (20.5)	15 (15.7)
Not working	128 (73.6)	54 (69.2)	74 (77.1)
**Chronic disease Status, n (%)**			
Yes	61 (35.1)	26 (33.3)	35 (36.5)
No	113 (64.9)	52 (66.7)	61 (63.5)
**Internet use, n (%)**			
Once to several times a week	21 (12.1)	8 (10.3)	13 (13.5)
Once a day	24 (13.8)	6 (7.7)	18 (18.8)
Several times a day	129 (74.1)	64 (82.1)	65 (67.7)
**Income, n (%)**			
A$ 104,000 +	18 (13.0)	9 (11.5)	9 (9.4)
A$ 65,000–103,999	20 (14.5)	10 (12.8)	10 (10.4)
A$ 41,000–64,999	37 (26.8)	11 (14.1)	26 (27.1)
A$ < 40,000	63 (45.7)	30 (38.5)	33 (34.4)
**Age in years, M (SD)**	69.53 (4.49)	69.88 (4.10)	69.12 (4.93)
**BMI, M (SD)**	29.41 (6.19)	29.34 (28.40)	29.46 (28.23)
**Internet self-efficacy, M (SD)**	44.39 (12.45)	43.74 (44.50)	44.92 (52.00)

Income missing (did not wish to disclose) *n* = 36. BMI missing *n* = 3

### Engagement with the Active for Life intervention

Table 2 presents average time on site, website visits, time reading tailored advice, module completion, time on exercise plans, exercise plan views, time on action plans, and action plan completion for the tailoring + Fitbit and tailoring only groups over the entire 12-week period. Results comparing the groups on each outcome are presented. Compared to the tailoring only group, the tailoring + Fitbit group had improved outcomes for time on site, website visits, time reading tailored advice, and module completion, however these differences were not significant. Conversely, compared to the tailoring only group, the tailoring + Fitbit group spent less time on the exercise plans (2.87(1,172), *p* = 0.06) and were less likely to complete 2 action plans (0.41 (0.27–0.63) *p* < 0.001).

**Table 2:** Engagement by intervention group

	**Total** M (SD)	**Tailoring + Fitbit** M (SD)	**Tailoring only** M (SD)	**Between group comparison** (F(df), p)
**Time on Site** (minutes [0–12 weeks]) *n* = 173^a^	99.85 (73.70)	103.46 (70.63)	96.90 (76.37)	0.34(1,172), *p* = .56
**Website visits** (total [0–12 weeks]) *n* = 173^a^	9.50 (6.08)	9.86 (6.05)	9.21 (6.12)	0.49(1,172), *p* = .49
**Time reading tailored advice** (minutes [0–12 weeks]) *n* = 173^a^	20.76 (18.60)	22.34 (17.83)	19.46 (19.20)	1.02(1,172), *p* = .31
**Module Completion** (min = 0, max = 6) *n* = 174	4.45 (2.17)	4.72 (2.04)	4.23 (2.25)	2.24(1,173), *p* = .14
**Time on exercise plans** (minutes 0–12 weeks) *n* = 173^a^	4.98 (12.44)	3.14 (6.92)	6.49 (15.46)	2.87(1,172) *p* = 0.06
**Exercise plan views** (total 0–12 weeks) *n* = 173^a^	2.52 (3.36)	2.06 (2.50)	2.89 (3.90)	3.59(1,172), *p* = 0.92
**Time on Action plans** (minutes 0–12 weeks) *n* = 173^a^	14.23 (14.47)	16.10 (14.63)	12.69 (14.24)	2.40(1,172), *p* = .12
**Action plan completion** (min = 0, max = 2), *n* = 174	**n (%)**	**n (%)**	**n (%)**	**Reference = Tailoring only** **OR (95%CI), p**
0	59 (33.9)	23 (29.5)	36 (37.5)	1
1	59 (33.9)	39 (50.0)	20 (20.8)	**1.70 (1.12–2.56), *p* = .012**
2	56 (32.2)	16 (20.5)	40 (41.7)	**0.41 (0.27–0.63) *p* < .001**

^a^Google analytics data were missing for 1 participant

**Fig. 1:**
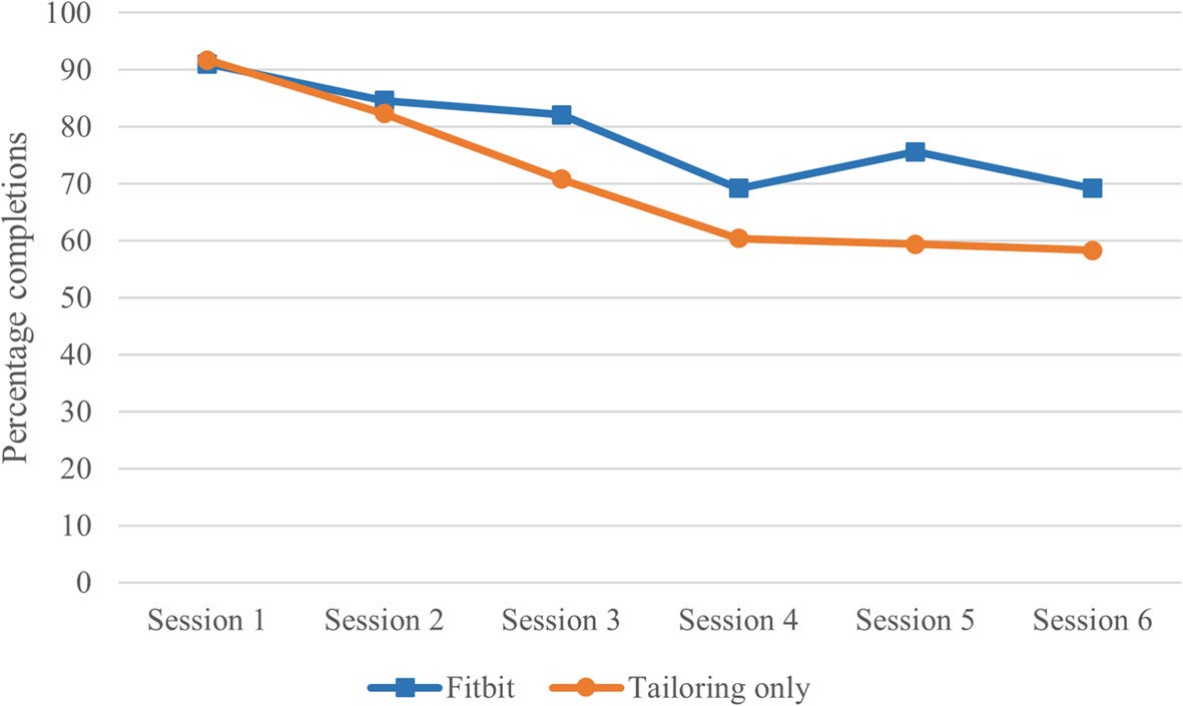
Module completions by intervention group (*n* = 174)

**Fig. 2:**
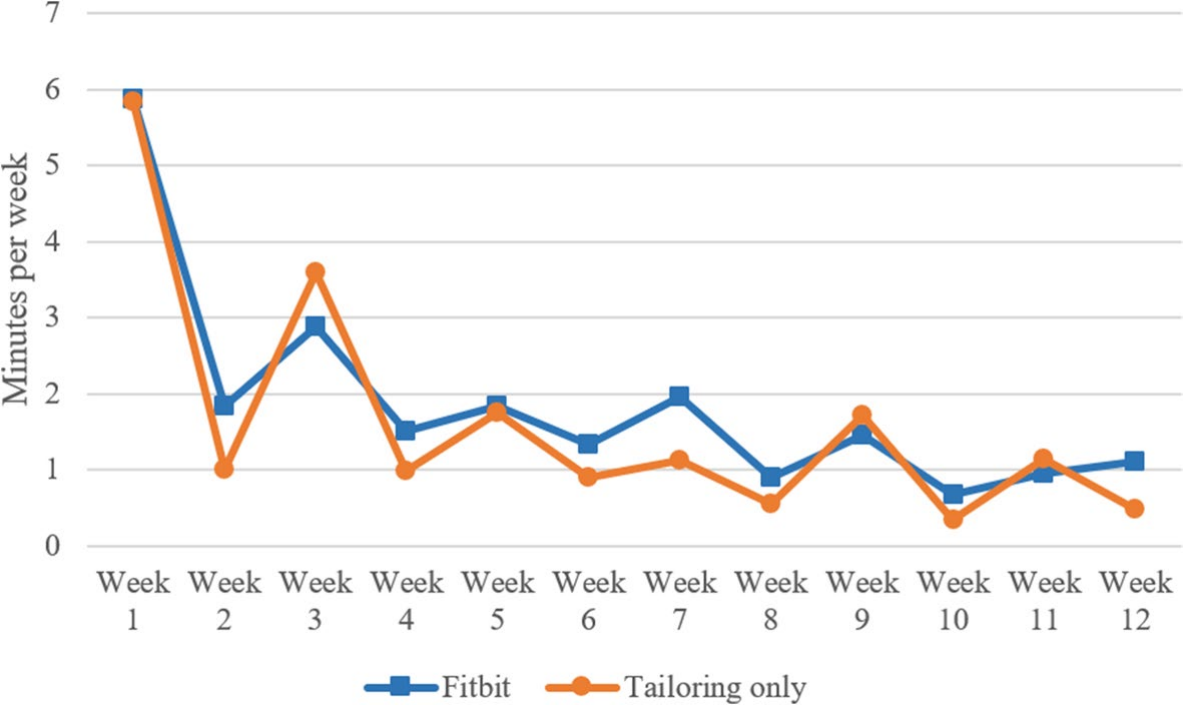
Time on tailored advice by week of intervention and intervention group (*n* = 173). Note. Intervention modules became available in week 1, 3, 5, 7, 9 and 11

Figure 1 presents percentage module completion by group for each module. The tailoring + Fitbit and tailoring only groups had a similar percentage of module completions for module 1 and 2, and the tailoring + Fitbit group had around a 10% higher completion rate for the following 4 modules. Figure 2 presents time spent on the tailored advice over the intervention period by group. Both intervention groups follow a similar trajectory.

#### Perceived usefulness and acceptability of the Active for Life intervention

Table 3 presents perceived usefulness and advice acceptability of the *Active for Life* intervention. Most participants rated the whole program as useful (82%) however, less than half (44%) rated the action planning tool as useful and just over half rated the exercise plans as useful (51%). These features were rated more useful in the tailoring only group with borderline significance. On average, participants gave a 5.7 out of 7 rating to the acceptability questions where 5 represents ‘somewhat agree’ and 6 represents ‘agree.’ Most participants agreed that the advice was interesting, credible, and easy to understand (> 90%). Most participants learned something new, found the advice personally relevant, used the advice to become more active and felt that it held them accountable to become more active (80–89%). A high number of participants changed their physical activity beliefs due to the program (71%) and about half thought that too much physical activity advice was provided (49%). No significant group differences for acceptability were observed.

**Table 3:** Usefulness and acceptability by group

	**Total n (%) agree**	**Tailoring + Fitbit n (%) agree**	**Tailoring only n (%) agree**	**Comparison** χ^2^, p
**Usefulness, *n* = 114**^**a**^				
Whole program	93 (81.6)	44 (80.0)	49 (83.3)	*p* = .67
Action Planning Tool	50 (43.9)	19 (34.5)	31 (52.5)	*p* = .05
Library of exercises	58 (50.9)	23 (41.8)	35 (59.3)	*p* = . 06
**Advice acceptability *n* = 115**				
The physical activity advice is interesting	105 (91.3)	51 (91.1)	54 (91.5)	*p* = .93
The physical activity advice is credible	108 (93.9)	52 (92.9)	56 (94.9)	*p* = .64
The physical activity advice is easy to understand	111 (96.5)	55 (98.2)	56 (94.9)	*p* = .33
Through the physical activity advice I learned something new about my own physical activity	98 (85.2)	45 (80.4)	53 (89.8)	*p* = .15
The physical activity advice is personally relevant	101 (87.8)	47 (83.9)	54 (91.5)	*p* = .21
Too much physical activity advice was provided	56 (48.7)	28 (50.0)	28 (47.5)	*p* = .78
I have used the physical activity advice to become more active	94 (81.7)	44 (78.6)	50 (84.7)	*p* = .39
I have changed my opinion about physical activity because of this program	82 (71.3)	41 (69.5)	41 (73.2)	*p* = .66
The physical activity advice held me accountable to become more active	98 (85.2)	47 (83.9)	51 (86.4)	*p* = .70
	**M (SD)**	**M (SD)**	**M (SD)**	**F (df), p**
Advice acceptability average rating (min = 1, max = 7) *n* = 115	5.69 (0.82)	5.62 (0.89)	5.75 (0.75)	F (1, 113) = 0.68, *p* = .41

^a^Percieved usefulness data were missing for 1 participant

#### Usability of the Active for Life website

Table 4 presents usability of the *Active for Life* intervention website. The average usability rating of the *Active for Life* website (M = 61) was below the established average SUS rating of other websites and systems (M = 68). A substantial number of participants in both groups felt that they needed to learn a lot to use the website (43%), needed support (38%), and that the website was complex (50%), cumbersome (44%) and inconsistent (44%). Yet, a large number of participants also indicated that the website was easy to use (84%), that most people could learn to use the website quickly (86%), and were confident in using the website (83%). Agreement for the statement ‘I found the website unnecessarily complex.’ was significantly lower in the Fitbit + tailoring group and the overall usability rating was significantly higher in the Fitbit + tailoring group. There were no other group differences on the individual usability questions. The total usability rating score was 7.5 out of 100 points higher in the tailoring + Fitbit group which was significant (*p* < 0.05).

**Table 4:** Usability by group

	**Total n (%) agree**	**Tailoring + Fitbit n (%) agree**	**Tailoring only n (%) agree**	**Comparison** χ^2^, p
**System Usability Scale *n* = 115**				
I think that I would like to use this website frequently	82 (71.3)	39 (69.6)	43 (72.9)	*p* = .70
I found the website unnecessarily complex	57 (49.6)	21 (37.5)	36 (61.0)	***p* = .012**
I thought the website was easy to use	96 (83.5)	49 (87.5)	47 (79.7)	*p* = .26
I think that I would need the support of a technical person to be able to use this website	44 (38.3)	17 (30.4)	27 (45.8)	*p* = .09
I found the various functions in this website were well integrated	90 (78.3)	42 (75.0)	48 (81.4)	*p* = .41
I thought there was too much inconsistency in this website	51 (44.3)	20 (35.7)	31 (52.5)	*p* = .07
I would imagine that most people would learn to use this website very quickly	99 (86.1)	50 (89.3)	49 (83.1)	*p* = .33
I found the website very cumbersome to use	51 (44.3)	21 (37.5)	30 (50.8)	*p* = .15
I felt very confident using the website	95 (82.6)	49 (87.5)	46 (78.0)	*p* = .18
I needed to learn a lot of things before I could get going with this website	50 (43.5)	21 (37.5)	29 (49.2)	*p* = .21
	**M (SD)**	**M (SD)**	**M (SD)**	**F (df), p**
Total SUS score, (min = 0, max = 100) *n* = 115	60.69 (13.56)	64.55 (13.59)	57.04 (12.58)	F (1, 114) = 9.50, ***p* = .003**

#### Satisfaction of the Active for Life intervention

**Fig. 3:**
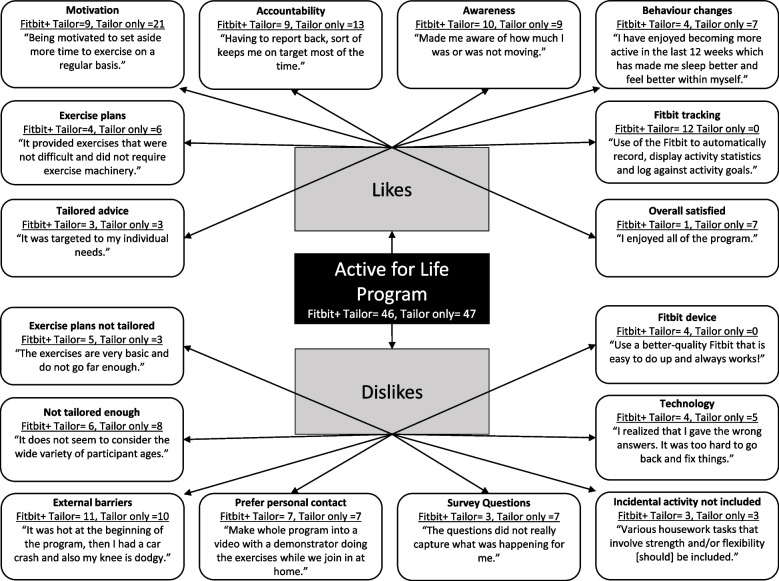
Satisfaction with the *Active for Life* program. Note: A total of 93 participants (Fitbit + tailoring = 46, tailoring only = 47) answered at least one open ended satisfaction question and are included in Fig. 3. The numbers for each category detail the number of participants from each group whose open-ended responses fit in that category (e.g., motivation)

Satisfaction with the *Active for Life* program was reported qualitatively through the 5 open ended questions. A total of 93 intervention participants responded to at least 1 of the 5 open ended questions and were included in the analysis. Findings are presented through a pen profile illustration (Fig. 3) under the themes of ‘likes’ and ‘dislikes.’

Under ‘likes’ participants mostly stated that they liked the motivational aspect (Fitbit + tailoring *n* = 9, tailoring only *n* = 21) and that it held them accountable (Fitbit + tailoring *n* = 9, tailoring only *n* = 13).

Having to report back, sort of keeps me on target most of the time (tailoring only, Female, 80).

Participants also stated that the program improved their awareness of their activity (Fitbit + tailoring *n* = 10, tailoring only *n* = 9), and helped them to change their behaviour (Fitbit + tailoring *n* = 4, tailoring only *n* = 7).

I have enjoyed becoming more active in the last 12 weeks which has made me sleep better and feel better within myself (Fitbit + tailoring, Male 66).

Participants in the Fitbit + tailoring group liked using the Fitbit to track their activity (Fitbit + tailoring *n* = 12, tailoring only *n* = 0). Some participants liked the exercise plans (Fitbit + tailoring *n* = 4, tailoring only *n* = 6) and a few liked how the physical activity advice was tailored to them (Fitbit + tailoring *n* = 3, tailoring only *n* = 3).

It was targeted to my individual needs (Fitbit + tailoring, Female, 72).

A small number mentioned that they were satisfied with the program overall (Fitbit + tailoring *n* = 1, tailoring only *n* = 7).

Under ‘dislikes’ participants mostly stated that external barriers such as weather, health, childcare, and moving house prevented them from participating in the program how they would have liked (Fitbit + tailoring *n* = 11, tailoring only *n* = 10).

It was hot at the beginning of the program, then I had a car crash and also my knee is dodgy (tailoring only, Female, 69).

Participants felt as though the tailored advice didn’t adequately take into account their situation and could have better considered factors such as age, health and external challenges (Fitbit + tailoring *n* = 6, tailoring only *n* = 8).

It does not seem to consider the wide variety of participant ages (Fitbit + tailoring, Male, 77).

Participants would have preferred personal contact (Fitbit + tailoring *n* = 7, tailoring only *n* = 7) and had some difficulties understanding and troubleshooting the technology including password, printing content, and accessing the strength exercises on the external website (Fitbit + tailoring *n* = 4, tailoring only *n* = 5).

I realized that I gave the wrong answers. It was too hard to go back and fix things (tailoring only, Female, 67).

Participants did not like the repetitive nature of the survey questions and felt as though there were not enough response options to capture their situation (Fitbit + tailoring *n* = 3, tailoring only *n* = 7).

The questions did not really capture what was happening for me (Fitbit + tailoring, Female, 65).

Participants (Fitbit + tailoring *n* = 5, tailoring only *n* = 3) also felt as though the exercise plans could have been better tailored to their exercise ability. Most of these participants indicated that they were too easy for them (Fitbit + Tailor *n* = 3, Tailor only *n* = 2).

The exercises are very basic and do not go far enough (tailoring only, Female, 67).

Some participants felt as though incidental exercise was not well integrated into the survey questions, tailored advice or exercise plan (Fitbit + tailoring *n* = 3, tailoring only *n* = 3).

Various housework tasks that involve strength and/or flexibility [should] be included (tailoring only, Female, 76).

A few Fitbit + tailoring participants had problems with their Fitbit device or did not like using the model of Fitbit device (Fitbit + tailoring *n* = 4, tailoring only *n* = 0).

#### Fitbit use, acceptability, and satisfaction

Tailoring + Fitbit participants reported at week 12 that on average they were using the Fitbit 14 h per day. Table 5 reports objective Fitbit Flex usage in tailoring + Fitbit participants. In the week prior to sessions 1–5 participants wore the device for 6 out of 7 days on average and most participants (87%-92%) wore the device on at least 5 days. Fitbit usage reduced slightly in the week prior to session 6 (final session) where participants wore the device for 5 out of 7 days on average and 78% wore the device on at least 5 days.

**Table 5:** Fitbit use

	Number of days used in week prior^a^	Participants wearing device for ≥ 5 days^a^
	**M (SD)**	**n (%)**
Session 1, Week 2 (*n* = 71)	5.75 (2.12)	62 (87.3)
Session 2, Week 4 (*n* = 66)	5.98 (2.00)	59 (89.4)
Session 3, Week 6 (*n* = 64)	6.19 (1.79)	59 (92.2)
Session 4, Week 8 (*n* = 54)	5.91 (2.00)	48 (88.9)
Session 5, Week 10 (*n* = 58)	6.12 (1.95)	52 (89.7)
Session 6, Week 12 (*n* = 54)	5.33 (2.81)	42 (77.8)

^a^Cut point ≥ 10 active minutes per day

Table 6 reports Fitbit Flex acceptability in tailoring + Fitbit participants. On average, participants gave a 6 out of 7 rating to the Fitbit acceptability questions which corresponds to an ‘agree.’ Most participants (89- 93%) agreed that the Fitbit increased their awareness of how active they were, helped them to meet their activity goals, made them more active and improved the user-friendliness of the advice. Fewer, but still a high percentage (80–82%) agreed that the Fitbit improved the value, credibility, and personal relevance of the advice. However less than half (40%) agreed that it was easy to connect and sync Fitbit data with the website.

**Table 6:** Fitbit acceptability

	**n (% Agree)**
Do you think the use of an activity tracker (Fitbit) in addition to the online physical activity advice helped in any of the following:	
- To improve the value of the web-based physical activity advice?	59 (80.8)
- To improve the credibility of the web-based physical activity advice?	59 (81.9)
- To improve the personal relevance of the web-based physical activity advice?	59 (81.9)
- To increase your awareness of how active you are?	66 (91.7)
- To meet your physical activity goals?	65 (89.0)
- To make you more active?	68 (93.2)
- To improve the user-friendliness of the physical activity advice?	68 (93.2)
It was easy to connect with and sync Fitbit data between the Active for Life website and the Fitbit website?	29 (39.7)
	**M (SD)**
Fitbit acceptability average rating (min= 0, max=7, n=72)	5.99 (0.80)

**Fig. 4:**
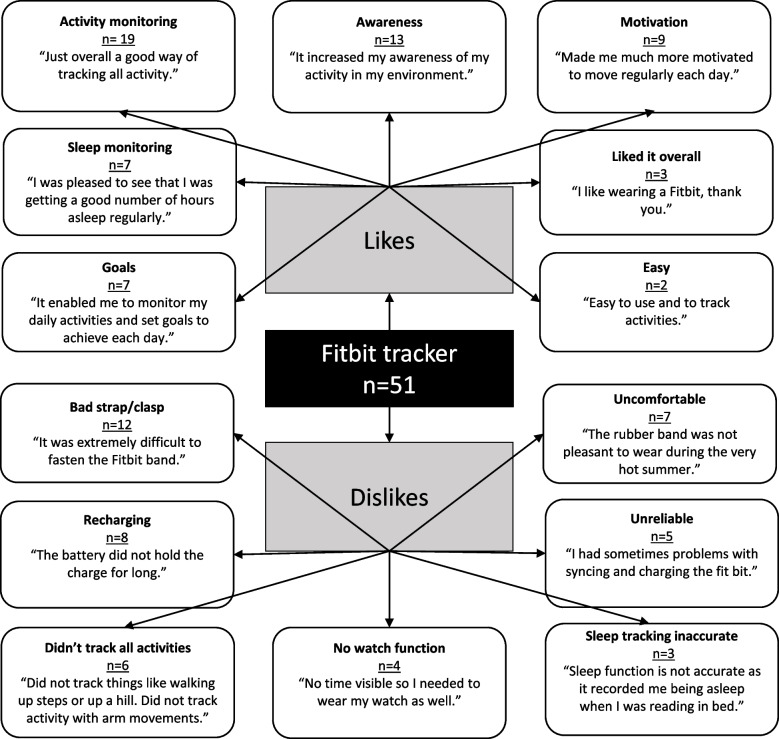
Satisfaction with the Fitbit tracker in the *Active for Life* program. Note: A total of 51 Fitbit + tailoring participants answered at least one open ended Fitbit satisfaction question and are included in Fig. 3. The numbers for each category detail the number of participants from each group whose open-ended responses fit in that category (e.g., goals)

Satisfaction with use of the Fitbit Flex tracker in *Active for Life* was reported qualitatively through the 3 open ended questions. A total of 51 Fitbit + tailoring participants responded to at least 1 of the 3 open ended questions and were included in the analysis. Findings are presented through a pen profile illustration (Fig. 4) under the themes of ‘likes’ and ‘dislikes.’

Under ‘likes’ Fitbit + tailoring participants mostly stated that they liked that the Fitbit allowed them to monitor their daily activity (*n* = 19) and how it improved their awareness of how much activity they were doing (*n* = 13).

It increased my awareness of my activity in my environment (Female, 75).

Participants found that wearing the Fitbit device and viewing their activity data helped to motivate them to move more (*n* = 9), and they liked the ability to set goals and receive daily feedback on progress towards their goal (*n* = 7).

It enabled me to monitor my daily activities and set goals to achieve each day (Male, 67).

Some participants also liked that the Fitbit monitored their sleep patterns (*n* = 7). Some participants mentioned that they liked the Fitbit overall (*n* = 3) and a couple of participants said that they found it easy to use (*n* = 2).

Under ‘dislikes’ Fitbit + tailoring participants mostly stated that they had problems with the Fitbit clasp and/or strap breaking or being difficult to do up (*n* = 12).

It was extremely difficult to fasten the Fitbit band (Female, 69).

Participants found that the device needed to be charged quite regularly (*n* = 8) and that it was uncomfortable to wear (*n* = 7). Participants found that the Fitbit device was unreliable as it sometimes didn’t charge, sync, or completely stopped working (*n* = 5). Some participants didn’t like that not all activities were tracked, or tracked accurately (e.g. walking up a hill, strength exercises, swimming) (*n* = 6).

Did not track things like walking up steps or up a hill. Did not track activity with arm movements (Female, 70).

Some participants mentioned that they would have liked a device which also had a watch function (*n* = 4) and found the sleep tracking inaccurate (*n* = 3).

## Discussion

### Active for Life program

The main aim of this study was to compare engagement, perceived usefulness, acceptability, usability, and satisfaction of the *Active for Life* intervention groups. Perceived usefulness of the whole program was reasonable with 82% of participants agreeing that it was useful. Website usage (100 min across 9.5 visits) in both groups were very good compared to similar studies in younger adults [10, 34, 35] (77–90 min total, 7.3–7.6 modules). Participants reported through the open-ended questions that they have good levels of satisfaction of the *Active for Life* program and that it helped motivate them, hold them accountable, improved their awareness and helped them to change their behaviour. The main dislikes were that external factors prevented them from making the most of the program and that they would prefer some personal contact. No web-based physical activity interventions with computer-tailored advice for older adults have reported usefulness, engagement, and satisfaction outcomes [7, 8], however other web-based physical activity interventions have been well received by older adults in terms of usefulness and acceptability [36]. Wallbank, Sherrington, Hassett, Kwasnicka et al. [36] found 83% of participants to recommend a non-tailored web-based physical activity program for women over 50 years. The current findings add to the literature by supporting the viability of a physical activity intervention with tailored advice for older adults.

### Tailored physical activity advice

The module completion rates (4.5 out of 6 modules) in both groups were very good compared to similar studies in younger adults [10, 34, 35] (2.9–5/8 modules). Acceptability of the physical activity advice was also good (7/9). Therefore, the lack of significant physical activity changes in the tailoring only participants presented in the main outcomes paper is unlikely to be due to the lack of engagement or acceptability of the tailored advice modules. The participants were engaged enough to complete most of the modules; however, this content was not enough to lead to significant physical activity changes. Perhaps different or additional content in the modules would have been more effective. However, the *Active for Life* program adhered to theory and evidence-based behaviour change techniques.

Whilst some participants mentioned in their open-ended responses that they liked the tailored advice, many felt as though the advice was not tailored enough, that the surveys to inform the advice did not capture their situation, and that the incidental activities they already do were not factored into the advice. This might partially explain why the tailored advice alone did not lead to improved physical activity changes. Future interventions could consider further personalising the advice to better factor in participants’ situations such as age, external barriers, and incidental activities. This could be done through personal contact to discuss the tailored advice, adding further variables to tailor the advice on, or through use of a machine learning based chatbot [37].

The high number of participants who agreed to the acceptability question ‘I thought that too much physical activity advice was provided’ (49%) is not in line with past studies evaluating tailored physical activity advice. These studies found only 14% [10] and 15% [32] of participants thought that too much advice was provided per session and 20% [32] of participants thought that too many modules were provided. This is interesting considering *Active for Life* had less modules of similar length compared to these programs. The other acceptability ratings of the tailored advice in *Active for Life* were good, making it unlikely that the advice seemed long because they were not satisfied with it. Whilst the reason for this high aggregated rating is not clear, it suggests that future programs providing tailored advice to older adults should ensure that the tailored advice is kept succinct.

### Fitbit to inform tailored physical activity advice

There were no differences between intervention groups for time reading advice or module completion. This is not consistent with the findings of Vandelanotte, Duncan, Maher, Schoeppe et al. [10] who found computer-tailored physical activity advice based on Fitbit data to have more module completions (4.4/8) than tailoring only physical activity advice (2.9/8) in adults 18 + years. Further, provision of a Fitbit Flex device did not improve the perceived usefulness of the program or acceptability of the tailored advice. It was anticipated that using objective Fitbit data to inform the advice would lead participants to more likely to agree that the advice taught them something new about their activity, was credible or help to hold them accountable in line with adults 18 + years in Vandelanotte, Duncan, Maher, Schoeppe et al. [10] who used the same Fitbit Flex device. The lack of differences between groups may be as the Fitbit integration with the tailored advice was not as well received by adults 65 + years of age compared to adults 18 + years of age. It could also be influenced by the overall high acceptability ratings and engagement leaving little room for improvements due to use of the Fitbit.

Use of the Fitbit Flex was good (87%-92% of participants at sessions 1–5), however this reduced slightly (78%) by the last session of the intervention (session 6, week 11). Despite the week 12 drop, usage remained higher than activity tracker usage previously observed in adults of all ages (60% at week 12) [38]. Fitbit Flex acceptability ratings (> 80%) were high, except for the question ‘it was easy to connect with and sync Fitbit data’ where only 40% agreed. The acceptability questions related to the Fitbit itself (e.g., ‘did the Fitbit make you more active’) were scored higher (89–93%) than the questions regarding acceptability of the Fitbit data informing the tailored advice (81%). Additionally, the responses to the open-ended questions demonstrated that participants liked using the Fitbit device itself as it monitored their activity which improved their awareness and motivation. Participants liked monitoring their sleep and setting goals directly with their Fitbit but did not discuss how it informed their tailored physical activity advice. Therefore, participants might have benefited from the Fitbit mostly as a separate program component rather than its integration with the *Active for Life* website to inform the tailored advice. This could explain why acceptability of the tailored advice was not improved in the Fitbit + tailoring participants. The high usage and high ratings of acceptability of the Fitbit itself is in line with past research which found high usage and acceptability of activity trackers to motivate older adults to increase their activity [16]. Although the use of the Fitbit did not improve the acceptability or effectiveness of the tailored advice intervention, these good Fitbit usage and acceptability results demonstrate that the Fitbit is a viable strategy to use in future interventions targeting older adults. Responses to the open-ended questions suggest that future interventions should consider using a reliable model of activity tracker which has an easy to do up clasp and a long battery life. Consideration should also be given to providing a device that more accurately tracks a range of activities (e.g., heart rate monitor and altimeter), that’s comfortable to wear and that doubles as a watch so two devices don’t need to be worn.

### Usability of the intervention website

The usability rating in both groups was under the average rating of 68 out of 100. The specific areas of concern were that the website was inconsistent, cumbersome, and complex and that participants felt they needed to learn a lot before using the website and needed help. The under average rating tends to be the case in e-health systems for older adults [39] and shows the need for developers of e-health systems for older adults to ensure these systems are specifically designed in line with older adults’ needs and preferences. Responses to the open-ended questions demonstrated that participants had a range of difficulties with the website technology including the password, printing content, and accessing the strength exercises on the external website. Usability could be improved by providing instructions or ‘how to’ videos for website functions including resetting passwords and printing content and by improving the way the exercises are integrated in the website to allow participants to seamlessly connect and access the exercise plans. System updates based on detailed usability testing has been shown to improve usability ratings of e-health systems for older adults [40] and should be included in the development process. This process should help to avoid perceived inconsistencies, complexity, and cumbersome nature of future e-health websites for older adults.

Consistent with a trial in participants 18 + years of age [10], the tailoring + Fitbit group rated the website usability significantly higher overall compared to the tailoring only group. When looking at the individual usability questions less Fitbit + tailoring participants found the website unnecessarily complex compared to the tailoring only participants, despite the added complexity of having to sync Fitbit data. The only difference between the websites was that the tailoring + Fitbit group synced their physical activity data from their Fitbit at each module which 60% of Fitbit + tailoring participants found challenging. Therefore, the higher usability ratings in the Fitbit + tailoring group may be as the website more generally seems easy to use compared to the Fitbit and syncing the Fitbit with the website.

### Action planning tool and exercise program

The action planning tool and strength, balance, and flexibility exercise programs were perceived as useful for about half of participants. Time on exercise plans and action plans were also low and less than a third completed two action plans. Many participants discussed the exercise plans in the open-ended satisfaction questions. Participants liked that there were exercise plans to follow but many felt as though they could have been better tailored to their abilities. Therefore, to improve perceived usefulness and engagement with the exercise plans, they could be better tailored to participants abilities. A short assessment could be carried out at the start of the program to provide a tailored plan to participants. Unlike the exercise plans, action plans were barely mentioned in the open-ended satisfaction questions. Other web-based health behaviour change programs in younger adults also found low use of action plans [32, 41, 42]. As action planning is associated with improved behaviour change, future web-based physical activity interventions should consider engaging ways to improve uptake of action planning tools. Time on exercise plans, completion of two out of two action plans and usefulness ratings of these features were improved in the tailoring only group and these group differences were borderline significant. This may be as participants in the Fitbit + tailoring group found the Fitbit a more helpful to engaging in physical activity and therefore rated the other features lower. It may also be that the Fitbit didn’t integrate well with these other features. For example, the exercise plans were made up of strength, balance and flexibility exercises that are not well tracked by the Fitbit. They may have therefore been more motivated to do activities such as walking to increase their activity as measured by the Fitbit.

### Strengths and limitations

The strengths of the study include randomisation to each intervention group, objective website and module completion measures, and qualitative data to add context and understanding to the qualitative findings. The study also had a good representation of people with a low household income (46% < A$40,000). A limitation includes the phone and face-to-face contact between participants and researchers, as well as monetary incentives which may have encouraged participants to engage with the intervention more than they would in a real-world scenario. In particular, the face-to face meeting with researchers at baseline may have helped to remove barriers to participation (e.g., syncing Fitbit to the website) and the website usability ratings and Fitbit acceptability ratings may not generalise to a real world scenario without hands on support. Lastly, Fitbit usage was measured by self-report and estimated from daily active minutes recorded by the Fitbit in the week prior to each *Active for Life* session. Future studies should consider recording continuous objective usage data (e.g., extract previous fortnight at each fortnightly session) over the entire intervention. Future studies could also consider using more advanced activity trackers to calculate wear time from heart rate data.

## Conclusions

Engagement, perceived usefulness, and satisfaction of the overall program were good. Engagement and acceptability of the tailored advice specifically were good despite the main outcomes paper reporting no physical activity changes observed in the tailored advice only participants. Participants explained that they wanted further personalisation of the tailored advice and/or personal contact. Fitbit integration did not improve the engagement or acceptability of the tailored advice, however participants engaged with and felt as though they benefited from using a Fitbit device. Usability ratings of the website were modest but highest in the Fitbit + tailored advice group. The action planning tool and exercise plans had a low engagement and perceived usefulness, and many participants felt as though the exercise plans were not well tailored to their abilities. Future web-based behaviour change interventions for older adults should ensure advice and exercise plans are well tailored to participants’ abilities, age and situation, consider including personal contact, better integrate activity tracker data with other intervention components, and ensure adequate website usability testing with the target group.

## Data Availability

The datasets used and/or analysed during the current study are available from the corresponding author on reasonable request.

## Abbreviations

MVPA: Moderate to Vigorous Physical Activity
SUS: System Usability Scale
BMI: Body Mass Index
ANOVA: Analysis of Variance

## References

[1] Bauman A, Merom D, Bull FC, Buchner DM, Fiatarone Singh MA (2016). Updating the Evidence for Physical Activity: Summative Reviews of the Epidemiological Evidence, Prevalence, and Interventions to Promote "Active Aging". Gerontologist.

[2] Australian Bureau of Statistics. National Health Survey: First Results, 2017–18 Canberra, Australia. 2019. https://www.abs.gov.au/statistics/health/health-conditions-and-risks/national-health-survey-first-results/2017-18. Accessed 5 Sept 2022.

[3] Australian Bureau of Statistics. Use of information technology by people with disability, older people and primary carers. Canberra, Australia. 2020. https://www.abs.gov.au/articles/use-information-technology-people-disability-older-people-and-primary-carers#:~:text=Older%20people%20(65%20years%20and,in%20the%20previous%203%20months. Accessed 5 Sept 2022.

[4] Jonkman NH, van Schooten KS, Maier AB, Pijnappels M (2018). eHealth interventions to promote objectively measured physical activity in community-dwelling older people. Maturitas.

[5] Muellmann S, Forberger S, Mollers T, Broring E, Zeeb H, Pischke CR (2018). Effectiveness of eHealth interventions for the promotion of physical activity in older adults: A systematic review. Prev Med.

[6] Broekhuizen K, Kroeze W, van Poppel MN, Oenema A, Brug J (2012). A systematic review of randomized controlled trials on the effectiveness of computer-tailored physical activity and dietary behavior promotion programs: an update. Ann Behav Med.

[7] Volders E, Bolman CAW, de Groot RHM, Verboon P, Lechner L (2020). The Effect of Active Plus, a Computer-Tailored Physical Activity Intervention, on the Physical Activity of Older Adults with Chronic Illness(es)-A Cluster Randomized Controlled Trial. Int J Environ Res Public Health..

[8] Van Dyck D, Herman K, Poppe L, Crombez G, De Bourdeaudhuij I, Gheysen F (2019). Results of MyPlan 20 on Physical Activity in Older Belgian Adults: Randomized Controlled Trial. J Med Internet Res..

[9] Stockwell S, Schofield P, Fisher A, Firth J, Jackson SE, Stubbs B (2019). Digital behavior change interventions to promote physical activity and/or reduce sedentary behavior in older adults: a systematic review and meta-analysis. Exp Gerontol.

[10] Vandelanotte C, Duncan MJ, Maher CA, Schoeppe S, Rebar AL, Power DA (2018). The Effectiveness of a Web-Based Computer-Tailored Physical Activity Intervention Using Fitbit Activity Trackers: Randomized Trial. J Med Internet Res.

[11] Oliveira SJ, Sherrington C, Zheng ERY, Franco RC, Tiedemann A. Effect of interventions using physical activity trackers on physical activity in people aged 60 years and over: a systematic review and meta-analysis. Br J Sports Med. 2019;54:1188-1194. 10.1136/bjsports-2018-100324 31399430

[12] Alley S, Schoeppe S, Guertler D, Jennings C, Duncan MJ, Vandelanotte C (2016). Interest and preferences for using advanced physical activity tracking devices: results of a national cross-sectional survey. BMJ Open.

[13] Brickwood KJ, Watson G, O'Brien J, Williams AD (2019). Consumer-Based Wearable Activity Trackers Increase Physical Activity Participation: Systematic Review and Meta-Analysis. JMIR Mhealth Uhealth.

[14] Vines J, Pritchard G, Wright P, Olivier P, Brittain K (2015). An Age-Old Problem: Examining the Discourses of Ageing in HCI and Strategies for Future Research. ACM Trans Comput Hum Interact.

[15] Tocci FL, Morey MC, Caves KM, Deberry J, Leahy GD, Hall K (2016). Are Older Adults Ready for Wireless Physical Activity Tracking Devices? A Comparison of Commonly Used Tracking Devices. J Am Geriatr Soc.

[16] Lyons EJ, Swartz MC, Lewis ZH, Martinez E, Jennings K (2017). Feasibility and acceptability of a wearable technology physical activity intervention with telephone counseling for mid-aged and older adults: a randomized controlled pilot trial. JMIR Mhealth Uhealth.

[17] Peels DA, Bolman C, Golsteijn RH, De Vries H, Mudde AN, van Stralen MM (2012). Differences in reach and attrition between Web-based and print-delivered tailored interventions among adults over 50 years of age: clustered randomized trial. J Med Internet Res.

[18] Alley SJ, van Uffelen J, Schoeppe S, Parkinson L, Hunt S, Power D (2022). The Effectiveness of a Computer-Tailored Web-Based Physical Activity Intervention Using Fitbit Activity Trackers in Older Adults (Active for Life): Randomized Controlled Trial. J Med Internet Res.

[19] Alley S, van Uffelen JG, Schoeppe S, Parkinson L, Hunt S, Power D (2019). Efficacy of a computer-tailored web-based physical activity intervention using Fitbits for older adults: a randomised controlled trial protocol. BMJ Open.

[20] Cardinal BJ, Esters J, Cardinal MK (1996). Evaluation of the Revised Physical Activity Readiness Questionnaire in older adults. Med Sci Sports Exerc.

[21] Department of Health. Physical Activity Recommendations for Older Australians (65 years and older). Australian Government 2019. https://www1.health.gov.au/internet/main/publishing.nsf/Content/health-pubhlth-strateg-phys-act-guidelines#npa%2065. Accessed 16 Dec 2020.

[22] Ajzen I (1988). Attitudes, personality, and behaviour.

[23] Bandura A (1986). Social foundations of thought and action: a social cognitive theory.

[24] Michie S, Ashford S, Sniehotta FF, Dombrowski SU, Bishop A, French DP (2011). A refined taxonomy of behaviour change techniques to help people change their physical activity and healthy eating behaviours. Psychol Health.

[25] Greaves CJ, Sheppard KE, Abraham C, Hardeman W, Roden M, Evans PH (2011). Systematic review of reviews of intervention components associated with increased effectiveness in dietary and physical activity interventions. BMC Public Health.

[26] Brooke J. SUS - a quick and dirty usability scale. . In: Jordan PW, Thomas B, Weerdmeester AL, McClelland AL, editors. Usability evaluation in industry. 189. 1st ed. London Taylor and Francis; 1996. p. 194.

[27] Brooke J. SUS - a quick and dirty usability scale. In: Jordan PW, Thomas B, Weerdmeester AL, McClelland AL, editors. Usability evaluation in industry. 189. London: Taylor and Francis; 1996. p. 194.

[28] Orstad SL, Gerchow L, Patel NR, Reddy M, Hernandez C, Wilson DK, et al. Defining Valid Activity Monitor Data: A Multimethod Analysis of Weight-Loss Intervention Participants' Barriers to Wear and First 100 Days of Physical Activity. Informatics (MDPI). 2021;8:39. 10.3390/informatics8020039 PMC9754231 36530339

[29] Finkelstein EA, Haaland BA, Bilger M, Sahasranaman A, Sloan RA, Nang EEK (2016). Effectiveness of activity trackers with and without incentives to increase physical activity (TRIPPA): a randomised controlled trial. Lancet Diabetes Endocrinol.

[30] Tudor-Locke C, Craig CL, Aoyagi Y, Bell RC, Croteau KA, De Bourdeaudhuij I (2011). How many steps/day are enough? For older adults and special populations. Int J Behav Nutr Phys Act.

[31] Eastin M, S. , LaRose R: Internet self efficacy and the psychology of the digital divide. J Comput-Mediat Commun. 2000;6. https://onlinelibrary.wiley.com/doi/full/10.1111/j.1083-6101.2000.tb00110.x.

[32] Schoeppe S, Duncan MJ, Plotnikoff RC, Mummery WK, Rebar A, Alley S (2021). Acceptability, usefulness, and satisfaction with a web-based video-tailored physical activity intervention: The TaylorActive randomized controlled trial. J Sport Health Sci.

[33] Ridgers ND, Timperio A, Brown H, Ball K, Macfarlane S, Lai SK (2018). Wearable Activity Tracker Use Among Australian Adolescents: Usability and Acceptability Study. JMIR Mhealth Uhealth.

[34] Eysenbach G (2005). The law of attrition. J Med Internet Res.

[35] Vandelanotte C, Short CE, Plotnikoff RC, Rebar A, Alley S, Schoeppe S (2021). Are web-based personally tailored physical activity videos more effective than personally tailored text-based interventions? Results from the three-arm randomised controlled TaylorActive trial. Br J Sports Med.

[36] Wallbank G, Sherrington C, Hassett L, Kwasnicka D, Chau JY, Phongsavan P (2022). Acceptability and feasibility of an online physical activity program for women over 50: a pilot trial. Transl Behav Med.

[37] To QG, Green C, Vandelanotte C (2021). Feasibility, Usability, and Effectiveness of a Machine Learning-Based Physical Activity Chatbot: Quasi-Experimental Study. JMIR Mhealth Uhealth.

[38] Hermsen S, Moons J, Kerkhof P, Wiekens C, De Groot M (2017). Determinants for Sustained Use of an Activity Tracker: Observational Study. JMIR Mhealth Uhealth.

[39] Vaziri DD, Aal K, Ogonowski C, Von Rekowski T, Kroll M, Marston HR (2016). Exploring user experience and technology acceptance for a fall prevention system: results from a randomized clinical trial and a living lab. Eur Rev Aging Phys Act.

[40] Cornet VP, Daley CN, Srinivas P, Holden RJ (2017). User-Centered Evaluations with Older Adults: Testing the Usability of a Mobile Health System for Heart Failure Self-Management. Proc Hum Factors Ergon Soc Annu Meet.

[41] van Genugten L, van Empelen P, Oenema A (2014). Intervention use and action planning in a web-based computer-tailored weight management program for overweight adults: randomized controlled trial. JMIR Res Protoc.

[42] De Cocker K, Cardon G, Vergeer I, Radtke T, Vandelanotte C (2019). Who Uses Action Planning in a Web-Based Computer-Tailored Intervention to Reduce Workplace Sitting and What do Action Plans Look Like? Analyses of the Start to stand Intervention among Flemish Employees. Appl Psychol Health Well Being.

